# Clinical outcomes and molecular typing of heterogenous vancomycin-intermediate Staphylococcus aureus bacteremia in patients in intensive care units

**DOI:** 10.1186/s12879-015-1215-2

**Published:** 2015-10-23

**Authors:** Han-Chung Hu, Kuo-Chin Kao, Li-Chung Chiu, Chih-Hao Chang, Chen-Yiu Hung, Li-Fu Li, Tsui-Ping Liu, Lee-Chung Lin, Ning-Hung Chen, Chung-Chi Huang, Cheng-Ta Yang, Jang-Jih Lu

**Affiliations:** Department of Respiratory Therapy, Chang Gung Memorial Hospital, Taoyuan, Taiwan; Department of Respiratory Therapy, Chang Gung University, College of Medicine, Taoyuan, Taiwan; Department of Thoracic Medicine, Chang Gung Memorial Hospital, Chang Gung University, College of Medicine, Taoyuan, Taiwan; Department of Laboratory Medicine, Chang Gung Memorial Hospital at LinKou, 5, Fu-Shin St., Kwei-Shan Taoyuan, 333 Taiwan; Department of Medical Biotechnology and Laboratory Science, Chang Gung University, Taoyuan, Taiwan

**Keywords:** MRSA bacteremia, hVISA, Intensive care units

## Abstract

**Background:**

*Staphylococcus aureus* is one of most common pathogens in humans. Methicillin-resistant *S. aureus* (MRSA) accounts for 64 % of *S. aureus* bacteremia isolated in intensive care units (ICUs), and heteroresistant vancomycin-intermediates *S. aureus* (hVISA) is a phenotype of MRSA. However, studies focusing on the hVISA impact on critically ill patients are scarce.

**Methods:**

This was a retrospective study conducted in a tertiary medical center from January 2009 to December 2010. All adult patients in ICUs with MRSA bloodstream infection were eligible. A modified population analysis profile and area under the curve method was applied to all isolates to confirm hVISA phenotype. Multilocus sequence typing (MLST), staphylococcal cassette chromosome *mec* (SCC*mec*) and the accessory gene regulator (*agr*) typing were performed individually. Clinical outcomes including in-hospital mortality, length of stay in intensive care unit and hospital after MRSA bacteremia of the patients were also analyzed.

**Results:**

A total of 48 patients were enrolled and 14 patients were confirmed to have the hVISA phenotype. The prevalence of hVISA was 29.2 %. There was no difference in the age, sex, comorbidity, Charlson’s comorbidity score and previous vancomycin therapy between the hVISA and VSSA groups. The hVISA group had a significantly higher in-hospital mortality than the VSSA group (13/14 versus 22/34; *p* = 0.046). All of the 14 hVISA patients had an MIC = 2 mg/L by E-test and this represented a significant association between high MIC and the development of hVISA (*p* < 0.001). MLST analysis showed all the isolates in the hVISA group were ST239, while ST239 (14/34; 41.2 %) and ST5 (12/34; 35.3 %) were predominant in the VSSA group (*p* = 0.007). A comparison of the survivor and non-survivor group showed that the hVISA phenotype (OR 11.8; 95 % CI 1.1–126.99; *p* = 0.042) and sequential organ failure assessment (SOFA) score (OR 1.39; 95 % CI 1.07–1.81; *p* = 0.014) were independent factors significantly associated with in-hospital mortality.

**Conclusions:**

Patients in ICUs with MRSA bacteremia may have a higher in-hospital mortality if they have the hVISA phenotype. SOFA score is also predictor of mortality.

## Background

*Staphylococcus aureus* is one of the most common pathogens in humans and may involve a variety of organs such as eyes [1], cardiovascular system [2], osterarticular system [3], skins and soft tissues, respiratory tract and circulatory system [4]. Methicillin-resistant *S. aureus* (MRSA) emerged several decades ago and is still a concern worldwide, especially in critical ill patients because of higher treatment costs and mortality rates [5]. According to a recent report, MRSA accounts for 64 % of *S. aureus* bacteremia isolated in intensive care units (ICUs) [6].

Vancomycin, a potent glycopeptide that has been used for more than 50 years, is still regarded as the main agent of treatment for MRSA. However, recent attention has focused on the emergence of isolates with decreased vancomycin susceptibility. The first isolates with reduced vancomycin susceptibility were detected in Japan in 1997 [7]. Since then heteroresistant vancomycin-intermediates *S. aureus* (hVISA) has been considered a phenotype of MRSA and has been reported throughout the world. These hVISA isolates are resistant subpopulations that are present in fully vancomycin susceptible *S. aureus* (VSSA) at a rate of 1 per 10^5^–10^6^ organisms [8, 9]. They are considered as the stage preceding the development of VISA and frequently occur in isolates with a minimum inhibitory concentration (MIC) within the susceptible range (≧2 mg/L by E-test) [10].

Previous study had reported the prevalence of hVISA to range from 0 % to 50 % [10]. The causes of such wide variance have to do with different geographic areas and different screening and detection methods. Until now there has been no standard method for early and easy detection of isolates with hVISA; rather, the most accepted and reproducible method for detecting hVISA is the modified population analysis profile (mPAP) and comparing the area under the curve (AUC) with the control strain Mu3 [11]. Because this method is labor-intensive, it is not routinely used in clinical practice. Infections caused by hVISA have been associated with vancomycin treatment failure, persistent bacteremia, and prolonged hospital length of stay [12–14]. However, the impact of hVISA bacteremia on mortality has yet to be determined. A previous study reported that patients infected with a certain genotype of hVISA were related to lower mortality [13], yet another study found that mortality rate was not affected at all [15]. Furthermore, prior research enrolled all patients with MRSA bacteremia regardless of the site of treatment and the severity of illness, which may be, excluded the genotype of isolates, causes of the different results between studies. And while there was one report finding that in critically ill patients, MRSA bacteremia may cause more acute renal failure and higher attributable mortality than methicillin-susceptible *S. aureus* (MSSA) bacteremia [5], studies focusing on hVISA impact on critically ill patients are scarce.

The aim of this study was to survey the prevalence and genotype of hVISA among patients in ICUs with MRSA bacteremia. The risk factors for hVISA genotype such as age, primary infection site, comorbidity or history of glycopeptide exposure were also analyzed. A secondary aim was to compared the clinical features and outcomes between patients with hVISA and vancomycin-susceptible *S. aureus* (VSSA) bacteremia. Finally, we investigated the independent predictors for in-hospital mortality between survivor and non-survivor.

## Methods

### Study design and patients

This was a retrospective study conducted in a tertiary medical center with a 3700-bed general ward and a 278-bed adult ICU in northern Taiwan. From January 2009 to December 2010, patients with MRSA bloodstream infection (BSI) and treated in ICUs were eligible for the study. The definition of MRSA BSI was patients with MRSA in blood cultures and met the Centers for Disease Control and Prevention (CDC) criteria for bloodstream infection. [16] Adult patients (aged ≧ 18 years) and single pathogen in blood culture were included. If there was recurrent MRSA bacteremia or persistent bacteremia (> 7 days), only the isolate of MRSA and the data of the first episode was analyzed. This study was approved by the Chang Gung Medical Foundation Institutional Review Board (103-4279B).

### Clinical data

Data collection from patient medical records included: age, gender, admission date, presence of co-morbidity, Charlson comorbidity score, sequential organ failure assessment (SOFA) score, primary site of infection, history of glycopeptide use in the previous 1 month, receipt of chemotherapy or other immunosuppressive therapy in the previous 1 month, and concomitant antibiotics used in this episode. The primary site of infection was determined by assessment of other MRSA-positive cultures at the time of the MRSA blood culture. Other immunosuppressive therapy included ≥10 mg/day prednisone or equivalent for more than 2 weeks or any dose of another immunosuppressant. Hospital-acquired infection was defined as the positive blood cultures obtained more than 48 h after admission. Hemodyalysis meaned patients ever receiving renal replacemen therapy, either regularly or acute episode, during the bacteremia treatment. Antibiotic treatment was considered adequate if the used drug was susceptible to the isolated MRSA. During the treatment course of MRSA bactereremia, if pateitns treated with norepinephrine treatment more than 5 μg per minute or an equivalent dose of other vasopressor for 4 h or more was defined as shock episode. The status at discharge (alive or deceased) was recorded. We also calculated the admission days to first culture, that meaned the numbers of days from admission to performing blood culture.

Outcomes assessment included in-hospital mortality, the length of ICU stay and hospital stay after MRSA bacteremia. In-hospital mortality means patients’ status at discharge were deceased. Length of ICU stay after MRSA bacteremia was the numbers of days from the first positive blood culture to be transferred to ward or death. Length of hospital stay after MRSA bacteremia was the numbers of days from the first positive blood culture to discharge or death. For the purpose of analysis, patients were stratified into survivor and non-survivor group according to the status of discharge (alive or deceased). Mortality was considered to be attributable to MRSA bacteremia if blood cultures were positive for MRSA at the time of death, or if death occurred before the resolution of the signs and symptoms of MRSA infection or if death occurred within 14 days after the onset MRSA bacteremia without another explanation [17].

### Microbiological and molecular methods

The MIC of vancomycin was determined by broth microdilution according to the Clinical and Laboratory Standards Institute (CLSI) guidelines [18] and the susceptibility breakpoint was ≦ 2 mg/L. The E-test (bioMérieux) was also performed according to the manufacturer’s instructions for all isolates. All isolates were further analyzed by the mPAP-AUC method as previously described [11], with hVISA being confirmed if the ratio of the AUC of MRSA isolates to the reference strain Mu3 (ATCC 700,698) was ≧ 0.9. The staphylococcal cassette chromosome *mec* (SCC*mec*) and the accessory gene regulator (*agr*) typing were analyzed with multiplex PCR as described elsewhere [19, 20]. The *agr* function was determined by δ-hemolysin activity, and *agr* dysfunction may result in a reduction or absence of of δ-hemolysin production. Detection of δ-hemolysin production by means of previously reported [21], briefly, the production of δ-hemolysin was measured by crossstreaking perpendicularly to *S. aureus* RN4220, which produces β-hemolysin and enhance blood lysis by δ-hemolysin. δ-Hemolysin produced by a test isolate causes an enhanced hemolysis in the area where the hemolysis zone of the test strain overlaps with that of RN4220. Therefore, the absence of synergistic hemolysis (δ-hemolysis negative) at the intersection of the streaks of RN4220 represents *agr* dysfunction. Multilocus sequence typing (MLST) was also performed for all isolates, as described by Enright et al. [22].

### Statistical analysis

Continuous variables were compared using Student’s *t*-test or the Mann–Whitney *U*-test and are presented as mean ± SD. Categorical variables were analyzed using the *χ*^2^ test or Fisher’s exact test as appropriate. Risk factors for in-hospital mortality were analyzed by using univariable analysis. The variables significantly associated with the outcome on analysis (*P* ≤ 0.2) were included in the multivariable analysis by applying a multiple logistic regression based on backward elimination of data. The Hosmer-Lemeshow goodness-of-fit test was used for calibration when evaluating the number of observed and predicted deaths in risk groups for the death probabilities. All statistical analyses were performed using SPSS, version 15.0 (SPSS, Inc, Chicago, IL, USA). All statistical tests were two-tailed and a *p* value of less than 0.05 was considered to be statistically significant.

## Results

During the study period, there were a total of 117 patients with MRSA bacteremia in our hospital and 48 patients fulfilled this study’s criteria. In the 69 excluded patients, 2 were VISA and others were treated at ward. Of the 48 isolates, 14 (29.2 %) harbored the hVISA phenotype. The baseline demographics and clinical outcomes were compared between the two groups and are shown in Table 1. There was no difference in the age, sex, comorbidity, Charlson comorbidity score and previous vancomycin therapy. The SOFA score represented the severity of illness when MRSA bacteremia developed and there was also no differentce between the hVISA and VSSA groups. The primary site of infection was different among the two groups, and we were unable to located the origin of infection for 7 patients (50 %) in the hVISA group while pneumonia was predominant (18/34; 52.9 %) in the VSSA group. The clinical outcomes between these two groups are also shown in Table 1. There was a trend of the hVISA group having a longer hospital stay in the days before the MRSA episode, although there was no statistical significance (44.5 ± 48.3 versus 27.0 ± 20.5; *p* = 0.08). Attributable mortality from MRSA bacteremia was 42.9 % (6/14) in the hVISA group, and this was not different from the VSSA group (14/34, 41.2 %) (*p* = 0.915). The number of days in the ICU and for hospital stay after this episode also showed no statistical difference between these two groups. However, the hVISA group had a significantly higher in-hospital mortality than the VSSA group (13/14 versus 22/34; *p* = 0.046).

**Table 1:** Demographic and clinical outcomes for intensive care patients with hVISA and VSSA bacteraemia^a^

	hVISA (*n* = 14)	VSSA (*n* = 34)	*p* value
Patient demographics			
Median age (years)	71.4 ± 16.9	69.7 ± 12.3	0.76
Male sex	9 (64.3)	18 (52.9)	0.47
Comorbidity			
Chronic renal disease	5 (35.7)	13 (38.2)	0.87
Solid organ malignancy	3 (21.4)	5 (14.7)	0.57
Hematological malignancy	1 (7.1)	1 (2.9)	0.508
Diabetes mellitus	4 (28.6)	14 (41.2)	0.412
Liver cirrhosis	4 (28.6)	5 (14.7)	0.263
Heart disease	1 (7.1)	8 (23.5)	0.186
Chronic pulmonary disease	1 (7.1)	1 (2.9)	0.508
Cerebrovascular accident	2 (14.3)	10 (29.4)	0.271
Primary site of infection			0.045
Catheter-related infection	1 (7.1)	6 (17.6)	
Pneumonia	6 (42.9)	18 (52.9)	
Surgical wound infection	0	3 (8.8)	
Other	0	3 (8.8)	
Unknown	7 (50)	4 (11.8)	
CRP	147.3 ± 11.3	132.6 ± 75.6	0.62
Albumin	2.5 ± 0.5	2.5 ± 0.6	0.82
Charlson comorbidity score	2.7 ± 1.6	3.1 ± 2.1	0.307
SOFA score	8 ± 3.2	8.4 ± 4.1	0.774
Prior vancomycin therapy	5 (35.7)	10 (29.4)	0.669
Adequate antibiotics treatment	3	12	0.279
Hemodialysis	7	14	0.403
Shock after infection	4	13	0.386
Outcomes			
In-hospital mortality	13 (92.9)	22 (64.7)	0.046
Attributable mortality by MRSA bacteremia	6 (42.9)	14 (41.2)	0.915
Length of stay in hospital after MRSA bacteremia (days)	32.6 ± 34.4	33.4 ± 49.3	0.96
Length of stay in ICU after MRSA bacteremia (days)	27.7 ± 31.5	21.2 ± 45.9	0.629
Admission days to first culture	44.5 ± 48.3	27.0 ± 20.5	0.083

^a^All values presented as number (%) or mean value ± SD unless otherwise indicated

*CRP* C-reactive protein

Table 2 shows the distribution of vancomycin MICs and genotype among our hVISA and VSSA isolates. All of the 14 hVISA patients had an MIC = 2 mg/L by E-test and this represented a significant association between a high MIC and the development of hVISA (*p* < 0.001). Genotype analysis using MLST indicated a significant difference between the two groups (*p* = 0.007): all isolates in the hVISA group werewas ST239 (sequence type 239) in hVISA group, while ST239 (14/34; 41.2 %) and ST5 (12/34; 35.3 %) were predominant in the VSSA group. In the SCC*mec* type analysis, most isolates were associated with type II and III (100 % in the hVISA and 79.4 % in the VSSA group), which is compatible with the hospital-acquired nature of the infection (all were hospital-acquired infection). In the hVISA group, 10 patients (71.4 %) had an *agr* dysfunction, which was significantly higher than that in the VSSA group (8.8 %; *p* < 0.001).

**Table 2:** Microbiological and genotypic characteristics of hVISA and VSSA bacteriaemia in ICU

	Isolation no. (%)		
	hVISA (*n* = 14)	VSSA (*n* = 34)	*p*
Vancomycin MIC			
(E test)			< 0.001
1 mg/L	0	6	
1.5 mg/L	0	18	
2 mg/L	14	10	
MLST			0.007
ST-239	14 (100)	14 (41.2)	
ST-5	0	12 (35.3)	
ST-59	0	5 (14.7)	
ST-45	0	2 (5.9)	
ST-1	0	1 (2.9)	
SCC*mec* type			0.095
II	5 (35.7)	17 (50)	
III	9 (64.3)	10 (29.4)	
IV	0	5 (14.7)	
V	0	2 (5.95)	
*agr* subgroup			0.4
1	11 (78.6)	20 (58.8)	
2	3 (21.4)	13 (38.2)	
3	0	1 (2.9)	
*agr* dysfunction	10 (71.4)	3 (8.8)	<0.001

Overall in-hospital mortality was 72.9 % (35/48) in our study. Table 3 presents the univariate analysis of multiple factors for mortality, which indicates that hVISA phenotype (Odds ratio [OR] 7.09; 95 % confidence interval [CI] 0.82-61.01; *p* = 0.046), SOFA score (OR 1.33; 95 % CI 1.05–1.68; *p* = 0.009) and received hemodialysis (OR 6.53; 95 % CI 1.26–33.9; *p* = 0.016) were shown to be associated with in-hospital mortality. Comorbidity of cerebrovascular accident (CVA) seemed to have a protective effect on the survival of these patients for unknown reasons. Other factors such as age, primary site of infection, shock episode, *agr* dysfunction and MIC did not appear to be significantly different between survivors and non-survivors. In a multivariate logistic regression analysis, SOFA score (OR 1.39; 95 % CI 1.07–1.81; *p* = 0.014) and hVISA (OR 11.8; 95 % CI 1.1–126.99; *p* = 0.042) were found to be independent predictors of in-hospital mortality (Table 4).

**Table 3:** Clinical features of patients with MRSA bacteraemia associated with survivor and non-survivor^a^

	Survivor (*n* = 13)	Non-survivor (*n* = 35)	*p* value
Patient demographics			
Median age (years)	76.5 ± 11.5	67.8 ± 18.2	0.114
Male sex	7	20	0.838
hVISA	1	13	0.046
Charlson comorbidity score	2.4 ± 1.4	3.2 ± 2.1	0.207
SOFA score	5.9 ± 3.0	9.1 ± 3.8	0.009
Hemodialysis	2	19	0.016
Comorbidity			
Chronic renal disease	3 (23.1)	15 (42.9)	0.208
Solid organ malignancy	2	6	0.885
Hematological malignancy	0	2	0.379
Diabetes mellitus	7	11	0.154
Liver cirrhosis	1	8	0.232
Heart disease	3	6	0.640
Chronic pulmonary disease	0	2	0.379
Cerebrovascular accident	7	5	0.005
Primary site of infection			0.72
Catheter-related infection	3	4	
Pneumonia	6	18	
Surgical wound infection	1	2	
Other	0	3	
Unknown	3	8	
CRP	139.5 ± 84.7	135.6 ± 88.5	0.897
Albumin	2.6 ± 0.8	2.4 ± 0.5	0.425
Prior vancomycin therapy	4	11	0.627
Adequate antibiotics treatment	3	12	0.48
Shock after infection	2	15	0.077
*agr* dysfunction	1 (7.7)	12 (34.3)	0.065
Vancomycin MIC (Etest)			0.111
1 mg/L	1	5	
1.5 mg/L	8	10	
2 mg/L	4	20	

^a^All values presented as number (%) or mean value ± SD unless otherwise indicated

*CRP* C-reactive protein

**Table 4:** Logistic regression analysis of clinical variables associated with in-hospital mortality

Parameter	Beta coefficient	Standard error	Odds ratios (95 % CI)	*P* value
SOFA score	0.329	0.134	1.39 (1.07–1.81)	0.014
hVISA	2.468	1.213	11.8 (1.1–126.99)	0.042
Constant	1.745	2.205	5.727	0.429

C.I.: confidence interval; In multivariable logistic regression, the Hosmer–Lemeshow test results (*χ*2 = 12.728, degrees of freedom = 8, *P* = 0.122)

## Discussion

This retrospective study analyzed 48 patients with MRSA bacteremia treated in ICUs, which is a group of patients seldom the focus of previous reports, and revealed the prevalence of the hVISA phenotype in our hospital was 29.2 %. The overall in-hospital mortality was 72.9 % (35/48) in this study, but the in-hospital mortality of the hVISA group rose to 92.9 % (13/14).

Previous studies have noted that the frequency of hVISA phenotype is associated with high vancomycin MIC, which means a higher hVISA rate when the MICs are higher [10, 13, 23]. All of the MICs of the hVISA isolates in our data were 2 mg/L by E-test, which is consistent with these reports. Prior research pointed out that the hVISA phenotype may cause glycopeptides treatment failure (defined as prolonged symptoms or persistent bacteremia) with a variable range [12, 24], and a meta-analysis study also revealed that hVISA infection had a 2.3 times greater failure rate compared to that of VSSA infection (95 % CI, 1.53 to 3.67) [25]. In our hospital, we do not routinely follow blood culture, and only do so when the patients have persistent fever or prolonged symptoms after the appropriate glycopeptide treatment; however, we did not observe the similar adverse effect caused by hVISA in our study.

Although the impact of the hVISA genotype on clinical outcomes has been extensively explored in previous studies, their results have been conflicting. For example, whereas Van Hal et al. recently found that hVISA in the ST239 MRSA strain was an independent predictor of survival [13], Park and colleagues reported that the most common genotype of hVISA in their hospital was ST5 and there was no obvious impact on patients’ outcomes [15]. In our study, all-cause mortality was significantly high in the hVISA isolates which were all of the ST239 strain. The inconsistent results between studies may be due to the different pathogen factors related to geographic variation, different case selection and different definitions of outcomes. Furthermore, the uncertainty of the conclusions also suggests that other possible causes, such as genetic factors, in addition to sequence type (ST) may affect an organism’s virulence factors and a patient’s clinical outcomes.

SCC*mec* type has been used to distinguish community-associated MRSA (CA-MRSA) from healthcare-associated MRSA (HA-MRSA) and type II and III are the more likely type in HA- MRSA strains [26] In previous studies, SCC*mec* II was the predominant genotype and associated with reducing vancomycin susceptibility and increasing the presence of hVISA [27, 28], but there no significant effect on outcomes such as vancomycin failure or persistent bacteremia were found in either study. Similarly, the SCC*mec* type did not produce similar adverse outcomes in our study, but hVISA isolates were discovered to more likely harbour SCC*mec* III (9/14; 64.3 %). The different genotype of SCC*mec* in studies also indicates the variation of microbiological characteristics in different areas, as was mentioned above.

The *agr* gene controls staphylococcal virulence and several metabolic pathways, with a number of studies showing that *agr* dysfunction may be related to persistent bacteremia, attenuated vancomycin bactericidal activity, vancomycin heteroresistance and the development of vancomycin-intermediate *S. aureus* (VISA) phenotype [29–31]. Schweizer et al. demonstrated that *agr* dysfunction was significantly associated with increasing mortality in severely ill patients [32]. In our study, the majority of hVISA isolates (10/14; 71.4 %) were *agr* dysfunction, while only being 8.8 % in the non-hVISA group. We also found that there was a trend of higher in-hospital mortality in the defective *agr* gene group (*p* = 0.065), although the figures did not reach statistical significance. The small sample size may have impacted on the results.

As mention earlier that *S.aureus* bacteremia is, particularly in ICU, a leading cause of death in nosocomial infection with mortality rate that is as high as 50 %. Blot et al. compared outcomes in critically ill patients with MSSA and MRSA bacteremia and demonstrated that MRSA bacteremia had a higher attributable mortality. Acute renal failure, age and length of mechanical ventilation were factors associated with mortality in this group of patients [5]. Van Hal et al. analyzed all of the severity levels of patients with MRSA bacteremia and concluded that age, severity of illness, and transit to ICU were independent predictors of 30-day mortality [13], but hVISA may reduce mortality in a ST239 genotype MRSA infection. However, since there has been scant published evidence on the effect of hVISA on the mortality of critically ill patients, we focused on this group of patients and analyzed the possible predictive factors of all causes mortality. The results showed that the overall mortality in our study was relative high, choosing the serious population only and selecting in-hospital mortality as aim may correlate to the poor ouctome. In multivariable analysis, the hVISA phenotype and severity of illness (SOFA score) were predictors of in-hospital mortality in ICU patients with MRSA bacteremia. This result indicated that the hVISA phenotype may bring a adverse outcomes in critically ill patients.

Naturally, there are limitations in our study. First, this was a retrospective study and performed in a single center, which means that data collection was restricted and incomplete for some factors, and our data only reflected the phenotype and genotype of MRSA in our institution. Second, the sample size was small because we concentrated on critically ill patients; different conclusions may have been reached in a multivariate analysis if we expanded the number of enrolled cases. We also lack some data such as the storage period of isolates before they were tested and the time to appropriate therapy, these variables may also impact the results. Despite these limitations, we believe this study can provide us with a better understanding of the impact of MRSA bacteremia in severely ill patients.

## Conclusions

From this study we found that the prevalence of hVISA was 29.2 % in our critically ill patients. This phenotype was associated with high vancomycin MIC within the susceptibility range and also the highly defective *agr* gene. Factors such as hVISA phenotype and SOFA score were predictors of in-hospital mortality. Further prospective, large scale studies are needed to further validate these findings.

## Abbreviations

MRSA: Methicillin-resistant *Staphylococcus aureus*
ICUs: Intensive Care Units
hVISA: heteroresistant Vancomycin-intermediates *Staphylococcus aureus*
VSSA: Vancomycin susceptible *Staphylococcus aureus*
MSSA: Methicillin-susceptible *Staphylococcus aureus*
SOFA: Sequential Organ Failure Assessment
CLSI: Clinical and Laboratory Standards Institute
SCC*mec*: Staphylococcal Cassette Chromosome *mec*
agr: Accessory Gene Regulator
MLST: Multilocus Sequence Typing
ST: Sequence Type
CVA: Cerebrovascular Accident
